# Predicting Treatment Response to Neoadjuvant Chemoradiotherapy in Rectal Mucinous Adenocarcinoma Using an MRI-Based Radiomics Nomogram

**DOI:** 10.3389/fonc.2021.671636

**Published:** 2021-05-24

**Authors:** Zhihui Li, Shuai Li, Shuqin Zang, Xiaolu Ma, Fangying Chen, Yuwei Xia, Liuping Chen, Fu Shen, Yong Lu, Jianping Lu

**Affiliations:** ^1^ Department of Radiology, RuiJin Hospital LuWan Branch, Shanghai Jiaotong University School of Medicine, Shanghai, China; ^2^ Department of Radiology, Changhai Hospital, Shanghai, China; ^3^ Scientific Research Department, Huiying Medical Technology Co., Ltd, Beijing, China; ^4^ Department of Radiology, RuiJin Hospital, Shanghai Jiaotong University School of Medicine, Shanghai, China

**Keywords:** rectal mucinous adenocarcinoma, neoadjuvant therapy, radiomics, magnetic resonance imaging, nomogram

## Abstract

**Objective:**

To build and validate an MRI-based radiomics nomogram to predict the therapeutic response to neoadjuvant chemoradiotherapy (nCRT) in rectal mucinous adenocarcinoma (RMAC).

**Methods:**

Totally, 92 individuals with pathologically confirmed RMAC administered surgical resection upon nCRT in two different centers were assessed retrospectively (training set, n = 52, validation set, n = 40). Rectal MRI was performed pre-nCRT. Radiomics parameters were obtained from high-resolution T2-weighted images and selected to construct a radiomics signature. Then, radiomics nomogram construction integrated patient variables and the radiomics signature. The resulting radiomics nomogram was utilized to assess the tumor regression grade (TRG). Diagnostic performance was determined by generating receiver operating characteristic (ROC) curves and decision curve analysis (DCA).

**Results:**

Six optimal features related to TRG were obtained to construct a radiomics signature. The nomogram combining the radiomics signature with age and mucin deposit outperformed the radiomics signature alone in the training (AUC, 0.950 *vs* 0.843, *p* < 0.05) and validation (AUC, 0.868 *vs* 0.719, *p* < 0.05) cohorts. DCA demonstrated a clinical utility for the radiomics nomogram model.

**Conclusions:**

The established quantitative MRI-based radiomics nomogram is effective in predicting treatment response to neoadjuvant therapy in patients with RMAC.

## Introduction

Rectal mucinous adenocarcinoma (RMAC) represents an uncommon subtype of rectal cancer (RC), histologically defined as adenocarcinoma with areas of extracellular mucin accumulation in >50% of the cancer (1). Compared with non-mucinous rectal adenocarcinoma, RMAC has poorer prognosis, which is associated with reduced survival and decreased pathological response to neoadjuvant chemoradiotherapy (nCRT) (2).

Therefore, accurate preoperative evaluation of tumor response to chemoradiotherapy is of high importance in long-term prognosis and treatment decision making in patients with locally advanced RC (3–5), which would make the treatment more personalized and effective. However, tumor regression grade (TRG) determination is only confirmed by postoperative pathology. At present, a consensus on the value of magnetic resonance imaging (MRI) in evaluating RC response to nCRT has been reached (2–6). A related clinical trial proposed the concept of MR tumor regression grade (mrTRG) mainly based on high-resolution T2-weighted imaging (T2WI), which reflects patient outcome (6). However, the current mrTRG classification lacks quantitative correlation with pathological regression grade and needs to compare images before and after treatment. Therefore, no reliable and accurate evaluation system has been developed to predict the therapeutic response to nCRT in RMAC.

With advances in high-throughput technological and analytical tools, radiomics combining many imaging features shows obvious advantages in providing important information about the tissue features that are inaccessible to the human eye (7–16). Indeed, mounting evidence indicates potential benefits for radiomics in predicting treatment response in RC over traditional imaging approaches (17–22). Nevertheless, the value of radiomics nomogram based on pre-nCRT MRI in predicting tumor regression in RMAC remains undetermined. Therefore, the present work aimed to build a radiomics nomogram and assess its performance in predicting therapeutic response to nCRT in RMAC.

## Materials and Methods

### Participants

All methods used in the present research had approval from the local Institutional Review Board (Committee on Ethics of Biomedicine, Changhai Hospital), who waived the requirement for informed consent due to a retrospective design.

First, 82 patients with pathologically confirmed RMAC who underwent rectal MRI and were administered surgical resection upon nCRT in Changhai Hospital were enrolled between January 2016 and December 2019, as the primary cohort. Next, 59 patients with the same inclusion criteria were enrolled between January 2017 and December 2020 in RuiJin Hospital LuWan Branch and assigned to the validation cohort.

**Figure 1:**
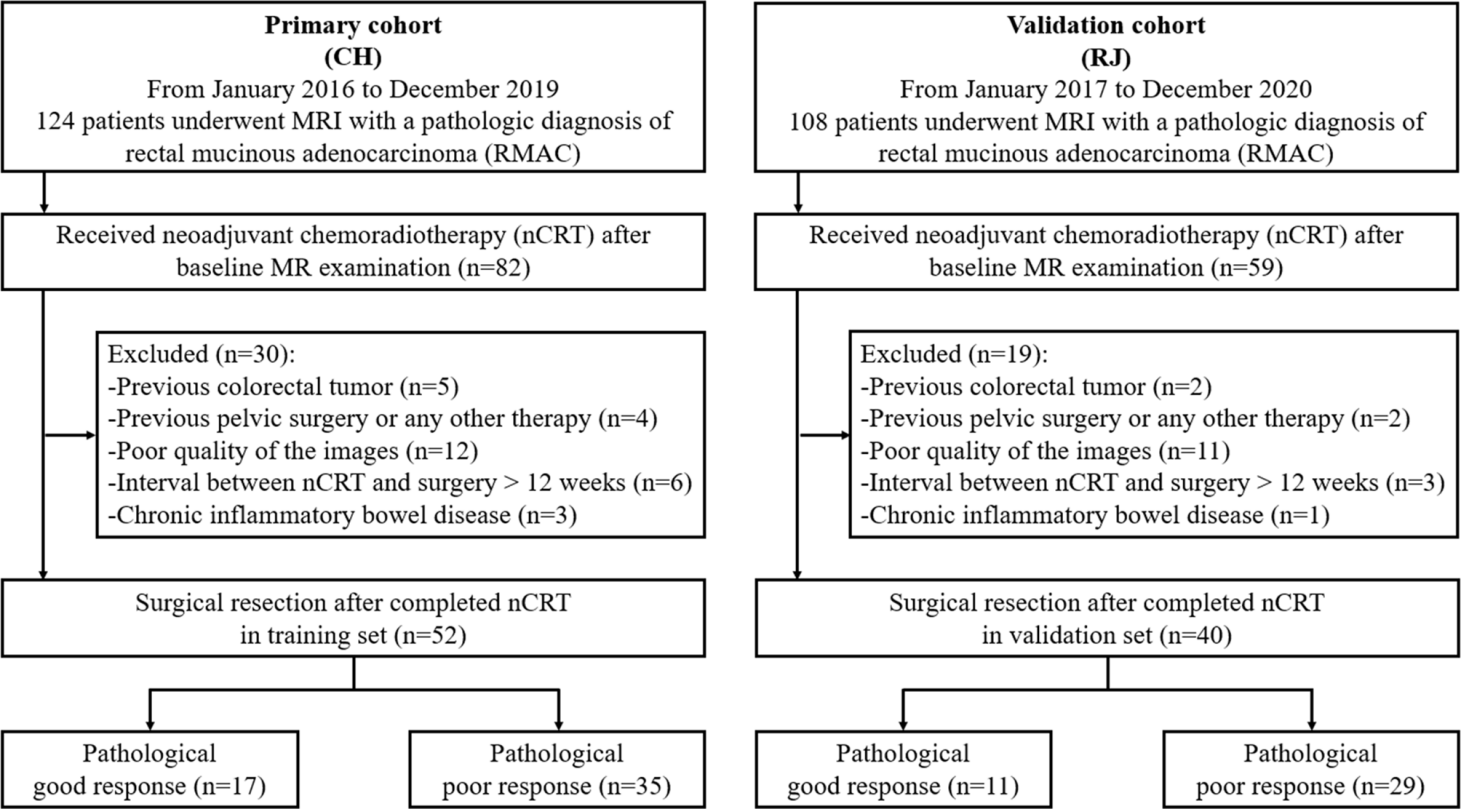
Study flowchart. CH, Changhai Hospital; RJ, RuiJin Hospital LuWan Branch.

Inclusion criteria were: (1) histologically confirmed RMAC with baseline MRI data; (2) baseline MRI within 14 days prior to neoadjuvant chemoradiotherapy (nCRT); (3) surgical resection after nCRT completion; (4) single focus tumor. Exclusion criteria were: (1) a history of previous colorectal tumor (n = 7); (2) previous pelvic surgery or any other treatment for cancer (n = 6); (3) poor quality of the images (n = 23); (4) interval between nCRT and surgery >12 weeks (n = 9); (5) chronic inflammatory bowel disease (n = 4). The study eventually included 52 and 40 cases in the training and validation sets, respectively (**Figure 1**).

Baseline patient data such as age, gender, BMI, pre-nCRT carcinoembryonic antigen (CEA) and carbohydrate antigen (CA19-9) were retrieved from medical records by a radiologist (SL, 10 years of experience).

### Image Acquisition and Conventional MR Imaging

Rectal MR examination was carried out on a 1.5 T or 3.0 T MR scanner using an abdominal phase array coil. All patients fasted for 4 h before the MR examination. Before scanning, intestinal cleaning was performed by enema administration with 20 ml of glycerin. Administration of raceanisodamine hydrochloride was waived for potential contraindications.

Routine rectal MR sequences, including high-resolution T2WI, were obtained. Oblique axial high-resolution T2WI was perpendicular to the main direction of the rectum comprising the lesion without fat suppression. Routine sequences, including sagittal T2WI, axial diffusion-weighted imaging (DWI, b value = 0, 1,000 s/mm^2^), axial T1-weighted imaging (T1WI), and gadolinium contrast-enhanced T1WI, were obtained in the sagittal, coronal, and axial planes. Details on the parameters applied for oblique axial high-resolution T2WI, which were used for radiomics model building, are shown in **Supplementary Table 1**.

Conventional MRI features, including the height of the tumor from the anal verge, tumor maximum thickness, MR-based T and N stages, mesorectal fascia (MRF), extramural vascular invasion (EMVI), and mucin deposit (MD) were assessed by two radiologists (SZ and SL, 10 years of experience each in MRI evaluation) in an independent fashion. In case of any discrepancies between the two examiners, a final decision was reached by consensus.

**Figure 2:**
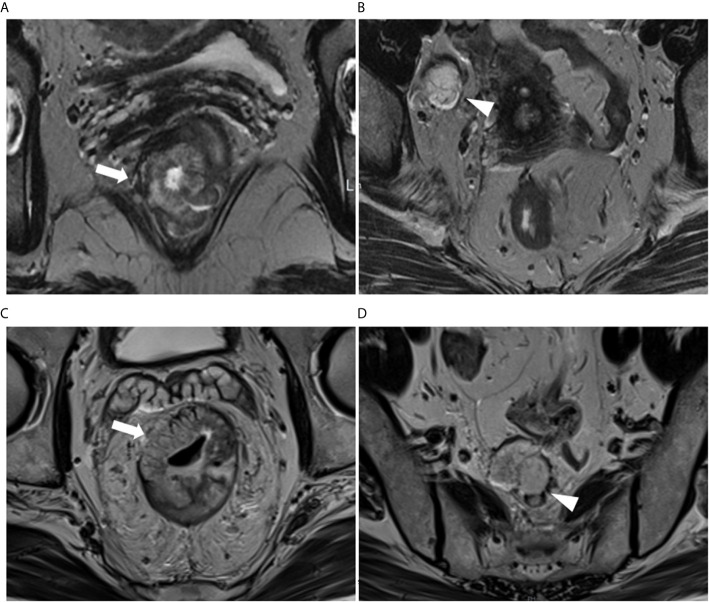
**(A, B)** A 61-year-old woman with RMAC in the lower rectum in the primary (training) cohort. **(A)** Oblique-axial T2WI demonstrating a lower rectal tumor with high signal intensity (arrows). **(B)** Right lateral pelvic tumor deposit with mucin content conspicuity (arrowhead). **(C, D)** A 65-year-old man with RMAC in the middle rectum in the validation cohort. **(C)** Oblique-axial T2WI demonstrating an RMAC (arrows). **(D)** Presacral mucin deposit demonstrating the similar presence of a primary rectal lesion (arrowhead).

Mucin deposit was defined as an atypical tumor deposit with mucinous component and short axes >5 mm in the mesorectum or perirectal tissues. On conventional MR imaging, mucin content within the regional deposit shows high-signal intensity on T2WI, demonstrates the same imaging features as that of the primary mucinous tumor. It can be clearly identified with T2WI by the similar presence of remarkable mucin pools with elevated signal intensity in primary rectal lesions (**Figure 2**). The short axes of the mucin deposits were measured.

### Neoadjuvant Chemoradiotherapy Treatment

The decision to administer nCRT was made by the chief surgeon, oncologist, and the patient. All patients received long-term pelvic radiation therapy with 45–50.4 Gy in 25–28 fractions, with concurrent administration of fluoropyrimidine (3–5). Radical resection surgery was initiated between 5 and 12 weeks after nCRT.

### Pathological Evaluation of the Therapeutic Response

Based on the National Comprehensive Cancer Network and American Joint Committee on Cancer (AJCC) TNM system (8th Edition) (23), all pathological tumor regression grades (TRGs) were obtained from surgical samples, with confirmation by histopathological evaluation. TRG was categorized as follows: 0 and 1, good response group (no residual viable malignant cells, only small cell clusters or single malignant cells); 2 and 3, poor response (residual malignant cells with substantial fibrosis, limited/no cancer cell death, or important residual tumor).

### Image Segmentation

The original baseline high-resolution T2WI DICOM images acquired pre-nCRT were imported into the Radcloud radiomics platform (Huiying Medical Technology, Beijing, China), based on the Image Biomarker Standardization Initiative (IBSI) standard. As the T2W images were acquired using different MR systems in both cohorts, image normalization was essential for all data to achieve homogeneity. Each image intensity was normalized to minimize MRI signal variation.

Regions of interest (ROIs) for all lesions were manually drawn slice-by-slice, which best fitted the tumor region in all samples on oblique axial high-resolution T2WI images. Next, volumes of interest (VOIs) were reconstructed based on these ROIs. Two radiologists with respectively 11 (ZL) and 8 years (FC) of experience in abdominal imaging independently performed image processing for all cases on the platform; they were blinded to patient information. Then, ZL repeated the segmentations for all cases one week later.

### Radiomics Features Selection and Radiomics Signature Building

Using the VOIs, radiomics features were extracted from each case with the above platform. Totally 1,409 features were categorized into four types: (1) first-order statistics, *e.g*., peak and mean values (with variance) quantitating voxel intensity distribution on MR images; (2) shape- and size-based features, *e.g.*, volume, surface area and spherical value, which reflect the 3D characteristics of the outlined area’s shape and size; (3) texture properties, including gray-level co-occurrence, run length, size zone and neighborhood gray-tone difference matrices, quantifying the selected area’s heterogeneity; (4) higher-order statistics, encompassing first-order statistics and texture features upon transformation (11–13).

In the training set, inter- and intraclass correlation coefficients (ICCs) were computed for evaluating the robustness of all features. Those showing both inter- and intra-observer ICCs ≥0.8 were applied for subsequent analysis. Then, the variance threshold algorithm, the select-K-best method and the least absolute shrinkage and selection operator (LASSO) algorithm were utilized for selecting optimal features.

Next, the selected features with their coefficients in the LASSO model were used for establishing a radiomics signature to determine a score for each patient (13). The radiomics signature’s performance was determined by the area under the receiver operator characteristic (ROC) curve (AUC) in both cohorts.

### Prediction Model of the Radiomics Nomogram

**Figure 3:**
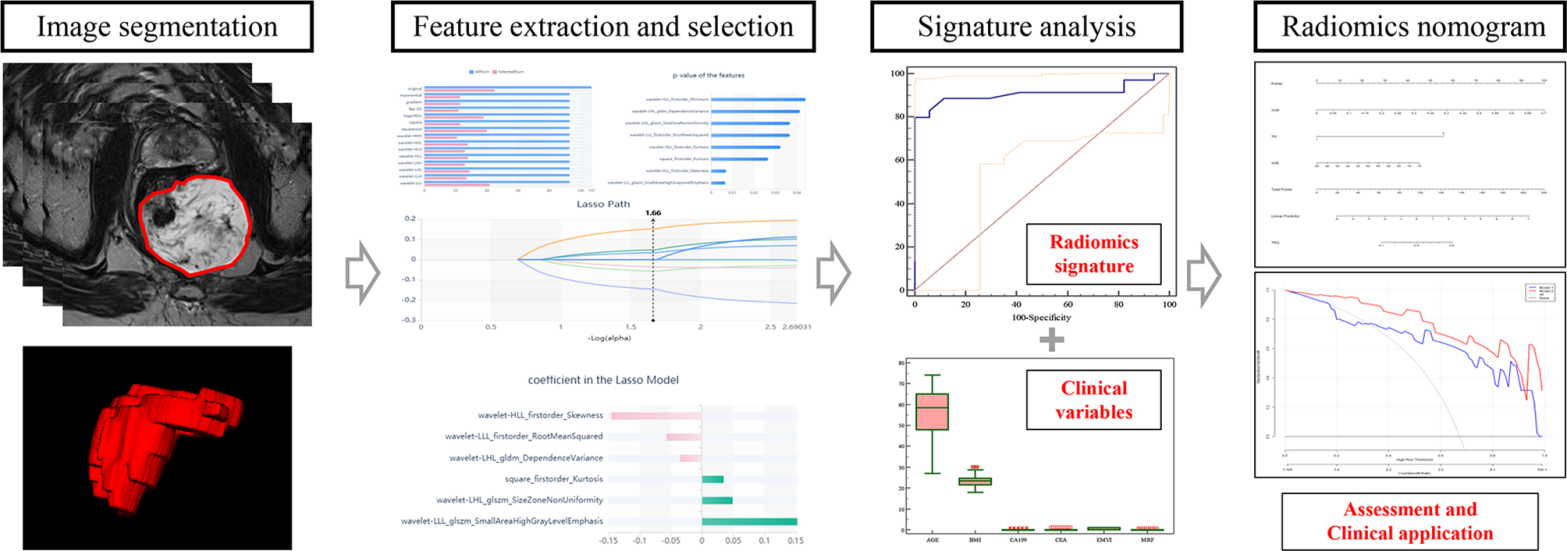
Workflow for building the radiomics nomogram.

Univariable analysis was carried out to examine differences in patient data and the radiomics signature between the good and poor response groups in the training set. Parameters with statistical significance were subsequently assessed by multivariable logistic regression analysis for developing a prediction model for TRG. This was followed by radiomics nomogram building. Then, the nomogram’s performance was assessed in both cohorts, respectively. The radiomics workflow is presented in **Figure 3**.

### Statistical Analysis

Continuous variables were assessed for normality by the Kolmogorov–Smirnov test, and compared by the t-test or Wilcoxon test. Qualitative variables were assessed by the Chi-square test or Fisher’s exact test. In the variance threshold method, 0.8 was the threshold, removing eigenvalues <0.8. In the select-K-best method, parameters with *p <*0.05 were utilized. In the LASSO model, the L1 regularizer served as the cost function, with an error of cross-validation of five and a maximum number of iterations of 1,000. ROC curves were obtained to evaluate both models for performance *via* AUC calculation in both cohorts, respectively. Sensitivity, specificity, accuracy, positive (PPV) and negative (NPV) predictive values, and positive (PLR) and negative (NLR) likelihood ratios were acquired. The Hosmer–Lemeshow test was utilized for goodness of fit estimation for the models. The DeLong test was carried out for evaluating differences between ROC curves. Decision curve analysis (DCA) was performed for determining the benefits of both models. R version 3.6.3 was utilized to analyze the nomogram. SPSS 20.0 (SPSS, USA) and MedCalc 19.6.1 were utilized for other data analyses. *P <*0.05 indicated statistical significance.

## Results

### Participant Features

Totally, 92 individuals (52 and 40 cases in the training and validation sets, respectively) were finally examined. The patient characteristics are listed in **Table 1** and were comparable in both cohorts. According to TRG by pathological examination after surgery, 17/52 patients (32.7%) were classified as good response in the training set, *versus* 11/40 (27.5%) in the validation set (*p* = 0.592).

**Table 1:** Demographic and clinical characteristics of the study patients.

Variables		Primary cohort	Validation cohort	*P* value
		n = 52 (%)	n = 40 (%)	
Gender (Male/Female)		40/12	27/13	0.314
Age (years)^*^		59 (27–74)	58 (33–72)	0.838
BMI (kg/m^2^)		23.4 ± 3.0	23.9 ± 2.9	0.423
Tumor height (cm)^* †^		4.0 (1–11)	4.5 (2–12)	0.511
Maximum thickness (cm)^†^		26.4 ± 10.3	22.5 ± 9.8	0.069
MR T stage	T1	0	0	0.735
	T2	6 (11.5)	4 (10.0)	
	T3	44 (84.6)	33 (82.5)	
	T4	2 (3.8)	3 (7.5)	
MR N stage	N0	6 (11.5)	5 (12.5)	0.967
	N1	30 (57.7)	22 (55.0)	
	N2	16 (30.8)	13 (32.5)	
MRF	Negative	42 (80.8)	32 (80.0)	0.927
	Positive	10 (19.2)	8 (20.0)	
EMVI	Negative	32 (61.5)	29 (72.5)	0.270
	Positive	20 (38.5)	11 (27.5)	
Mucin deposit	Negative	38 (73.1)	31 (77.5)	0.627
	Positive	14 (26.9)	9 (22.5)	
Pre-nCRT CEA	<5 ng/ml	41 (78.8)	35 (87.5)	0.278
	>= 5 ng/ml	11 (21.2)	5 (12.5)	
Pre-nCRT CA19-9	<37 U/ml	47 (90.4)	34 (85.0)	0.430
	>=37 U/ml	5 (9.6)	6 (15.0)	

MRF, mesorectal fascia; EMVI, extramural vascular invasion; CEA, carcinoembryonic antigen; CA19-9, carbohydrate antigen 19-9.

*Median (range).

^†^Measured by MRI.

In high-resolution T2WI without fat suppression, all RMACs had tumors containing a certain amount of high signal intensity mucin components (mucin pool). The mean short axes of the mucin deposits were 10.471 ± 3.804 mm in the training set and 12.567 ± 6.710 mm in the validation set (*p* = 0.061).

### Radiomics Features

**Figure 4:**
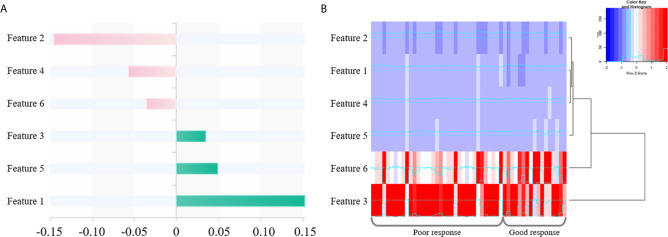
The selected six radiomics features. **(A)** Coefficients in the LASSO model. **(B)** A cluster analysis chart showing values for various radiomics features calculated for different responses to nCRT.

Totally 1272/1409 (90.3%) radiomics features had both inter- and intra-observer ICCs ≥0.8 in the training set, and were applied for subsequent analysis. Finally, six optimal features were obtained with the LASSO algorithm (**Table 2** and **Figure 4**) for radiomics signature (RS) construction.

**Table 2:** The selected radiomics features.

No.	Radiomics feature	Radiomics class	Filter
1	small area high gray level emphasis	GLSZM	wavelet-LLL^*^
2	skewness	first-order	wavelet-HLL^*^
3	kurtosis	first-order	square
4	root mean squared	first-order	wavelet-LLL^*^
5	size zone nonuniformity	GLSZM	wavelet-LHL^*^
6	dependence variance	GLDM	wavelet-LHL^*^

GLSZM, gray level size zone matrix; GLDM, Gray Level Dependence Matrix.

*The wavelet transform decomposes the tumor area image into low-frequency components (L) or high-frequency components (H) in the x, y, and z axes.

### Logistic Regression Analysis

**Figure 5:**
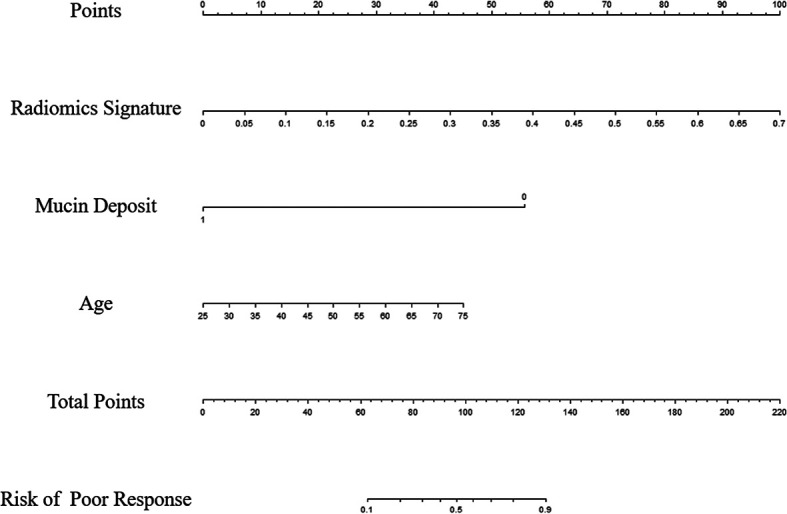
Radiomics nomogram developed in the training set for the prediction of poor response, based on radiomics signature, age and mucin deposit.

Univariable analysis revealed that the RS, age and mucin deposit (MD) were associated with TRG in human RMAC (**Table 3**). Then, radiomics nomogram building utilized a multivariable logistic regression analysis of chosen risk factors (age, OR = 1.060; MD, OR = 0.027) and RS (OR = 10,339.233) for the development of a predictive model for tumor response assessment (**Table 3** and **Figure 5**). Regression formula: prediction probability = −4.108 − 3.607 * MD + 9.244 * RS + 0.058 * AGE. The Hosmer–Lemeshow test showed a statistically favorable calibration of the nomogram model in both cohorts (*p* = 0.867 and *p* = 0.920, respectively).

**Table 3:** Logistic regression analyses of predicting tumor regression grade.

Variables	Univariate		Multivariate	
	OR (95% CI)	*P*	OR (95% CI)	*P*
Gender	1.615 (0.375–6.951)	0.519	NA	NA
Age	1.077 (1.015–1.142)	0.014	1.060 (0.974–1.154)	0.179
BMI	0.880 (0.717–1.081)	0.224	NA	NA
Tumor height	0.984 (0.741–1.307)	0.912	NA	NA
MR T stage	5.466 (0.970–30.793)	0.054	NA	NA
MR N stage	1.181 (0.462–3.017)	0.728	NA	NA
MRF	2.500 (0.611–10.228)	0.204	NA	NA
EMVI	1.704 (0.523–5.549)	0.376	NA	NA
CEA	1.383 (0.316–6.048)	0.667	NA	NA
CA19-9	1.422 (0.215–9.428)	0.715	NA	NA
Mucin deposit	0.090 (0.022–0.374)	0.001	0.027 (0.003–0.267)	0.002
Radiomics signature	3044.784 (32.395–286178.802)	0.001	10339.233 (17.241–6200476.598)	0.005

OR, odds ratio; NA: not available; MRF, mesorectal fascia; EMVI, extramural vascular invasion; CEA, carcinoembryonic antigen; CA19-9, carbohydrate antigen 19-9.

### Predictive Model

**Figure 6:**
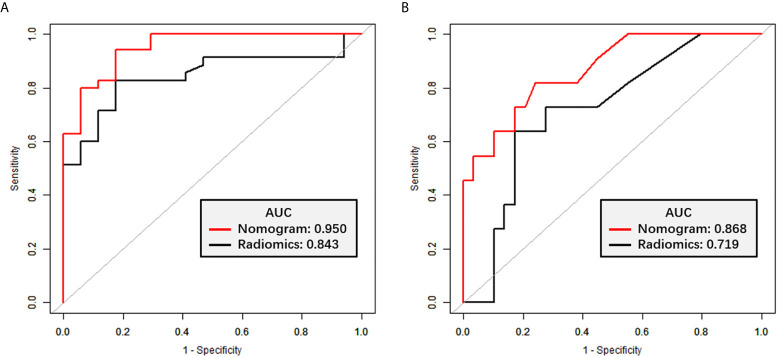
ROC curves for TRG classification. **(A)** ROC curves for the radiomics signature and nomogram models in the training set. **(B)** ROC curves for the radiomics signature and nomogram models in the validation set.

**Figure 7:**
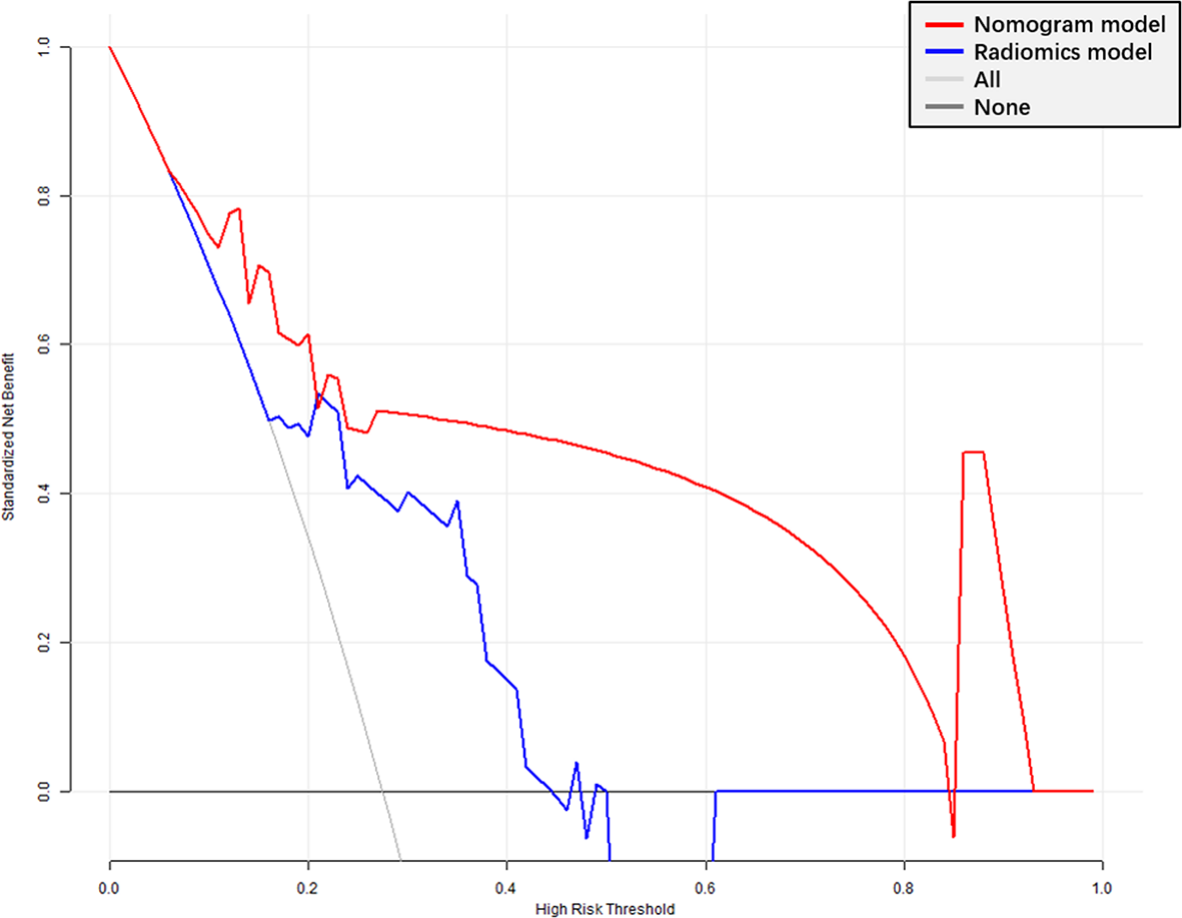
Decision curve analysis (DCA) of the radiomics signature and nomogram models. X-axis, probability of poor response to nCRT; Y-axis, net benefit. Black line, all patients assumed to be good responders; gray line, all patients considered poor responders. The nomogram model had enhanced net benefit compared with the radiomics signature in almost all threshold probabilities.

The radiomics signature had favorable predictive performance for treatment response (good *vs*. poor response), with AUCs of 0.843 and 0.719 in the training and validation cohorts, respectively. The AUCs of the radiomics nomogram were 0.950 and 0.868 in the training and validation sets, respectively. In both sets, the nomogram model had higher AUCs compared with the radiomics model alone. The DeLong test showed a significant difference (*p =* 0.037 and *p* = 0.042, respectively). Details are shown in **Table 4** and **Figure 6**. The decision curves demonstrated that in the validation cohort, the nomogram model showed a greater advantage compared with the radiomics signature at a threshold probability of 0.10–0.85 (**Figure 7**).

**Table 4:** ROC analysis of the prediction models in both cohorts.

	Primary cohort		Validation cohort	
	Radiomics	Nomogram	Radiomics	Nomogram
**AUC**	0.843	0.950	0.719	0.868
**95% CI**	0.734–0.952	0.893–1.000	0.546–0.893	0.746–0.991
**Specificity**	0.823	0.823	0.828	0.759
**Sensitivity**	0.829	0.943	0.636	0.818
**Accuracy**	0.827	0.904	0.775	0.775
**PLR**	4.695	5.343	3.691	3.390
**NLR**	0.208	0.069	0.439	0.240
**PPV**	0.906	0.917	0.583	0.562
**NPV**	0.700	0.875	0.857	0.917
***P***	0.037		0.042	

AUC, area under the curve; PLR, positive likelihood ratio; NLR, negative likelihood ratio; NPV, negative predictive value; PPV, positive predictive value.

## Discussion

Here, we developed and validated a radiomics nomogram model for predicting tumor response to treatment in RMAC. Integrating age, mucin deposit and a radiomics signature to generate a radiomics nomogram was efficient in predicting histologic tumor regression.

Compared with common rectal adenocarcinoma, RMAC has poorer differentiation and is associated with young age, prone to metastasis, and poor response to nCRT (2), due to their molecular differences compared to classical rectal adenocarcinoma (24, 25).

Several studies have demonstrated that the MRI-defined mucin pool in primary tumor before treatment independently predicts poor response to nCRT (5, 26–28). In post-nCRT MRI, the presence of mucin pools may be associated with better tumor regression (29–31). However, the significance of MRI-defined mucin deposit has not been previously reported by radiologists. Our results showed that mucin deposit was an independent image predictor of tumor response. The MRI-defined mucin deposit pattern prior to treatment appears as a high signal intensity deposit on T2WI scans, representing a biomarker for good response to preoperative chemoradiotherapy in RMAC. Our hypothesis is that the appearance of a mucin deposit suggests the rectal mucinous adenocarcinoma may contain more acellular mucin components rather than tumor components. On a molecular level, numerous aberrations have been described in the mucinous phenotype (32). There may be different molecular changes under different conditions.

In the current study, MRI features assessed by radiologists including MR T and N stages, MRF and EMVI were not associated with TRG in univariable analysis. This may be due to the relatively small sample size and non-quantitative subjective features on rectal MRI scans. Compared with traditional approaches of imaging diagnosis, radiomics as a novel imaging tool can significantly improve tumor diagnosis, grading, staging and prognosis prediction, to facilitate treatment planning (7–10). In this study, we automatically extracted 1409 radiomics features from pre-nCRT T2WI scans to comprehensively reflect the image phenotype of RMAC. After feature selection, six optimal features were chosen for developing a radiomics signature to preoperatively predict TRG, which exhibited favorable discrimination in both cohorts.

Next, three variables, including the radiomics signature, age, and mucin deposit, were integrated for developing a radiomics nomogram with increased discriminatory efficiency in both cohorts. The obtained radiomics nomogram represents a visualization tool for prognosis prediction in RMAC. In the current study, the radiomics signature and nomogram models were compared. The radiomics nomogram exhibited a higher predictive performance and provided enhanced net benefits in virtually all threshold probability ranges in DCA compared with the radiomics model alone (*p* < 0.05) for TRG prediction based on T2W images before treatment. Additionally, we applied an external validation cohort from another independent institution to confirm the discrimination efficacy of radiomics nomogram, which showed a favorable calibration in both cohorts. The above findings indicated the integrated radiomics nomogram has a potential to guide clinical practice. Identifying individuals with elevated odds of poor response preoperatively could help reassess the need for nCRT. If a patient prognostically had a poor response by the visualized and individualized nomogram, who may not benefit from conventional nCRT, he/she would require further treatment, such as epidermal growth factor receptor (EGFR) inhibitors and immune checkpoint inhibitors (ICIs) treatment.

The limitations of the current study should be mentioned. First, because of stringent inclusion criteria, the sample size was relatively small in this retrospective trial. However, external validation was performed with an independent cohort. Therefore, large multicenter studies are required to reduce the impact of data bias on model accuracy. Secondly, VOIs were manually rather than semi-automatically/automatically delineated, making it difficult to avoid subjective errors, and not suitable for large-scale data processing (33). Finally, this study did not include relevant molecular biological indicators. “Radio-genomics” taking into account radiomics and genomics features represents an emerging prognostic approach (34), which deserves further investigation.

## Conclusion

Overall, using high-resolution T2W images before neoadjuvant chemoradiotherapy, quantitative radiomics signature and nomogram models were built. This non-invasive approach can be applied for predicting tumor response to nCRT in RMAC; specifically, the combined nomogram model yielded enhanced clinical benefits compared with the radiomics signature alone in treatment decision making.

## Data Availability Statement

The original contributions presented in the study are included in the article/**Supplementary Material**. Further inquiries can be directed to the corresponding authors.

## Ethics Statement

All methods used in the present research had approval from the local Institutional Review Board (Committee on Ethics of Biomedicine, Changhai Hospital), who waived the requirement for informed consent due to a retrospective design. Written informed consent for participation was not required for this study in accordance with the national legislation and the institutional requirements.

## Author Contributions

JL, YL, and FS conceived the project. XM and FC acquired the data. LC, SL, and SZ analyzed and interpreted the patient data regarding radiomics features. YX performed statistical analyses and feature extraction. ZL was a major contributor in writing the manuscript. All authors contributed to the article and approved the submitted version.

## Funding

The present study was supported by the Project of the Action Plan of Major Diseases Prevention and Treatment (2017ZX01001-S12) and the Special Project of Integrated Traditional Chinese and Western Medicine in General Hospitals of Shanghai (ZHYY-ZXYJHZX-201901). The funders developed the main idea and designed the study.

## Conflict of Interest

YX was employed by the company Huiying Medical Technology Co., Ltd.

The remaining authors declare that the research was conducted in the absence of any commercial or financial relationships that could be construed as a potential conflict of interest.

## Abbreviations

RMAC, Rectal mucinous adenocarcinoma; TME, Total mesorectal excision; ICC, Intraclass correlation coefficient; T2WI, T2 weighted imaging; VOI, Volume of interest; LASSO, Least absolute shrinkage and selection operator; nCRT, Neoadjuvant chemoradiotherapy; TRG, Tumor regression grade; OR, Odds ratio; DCA, Decision curve analysis.
