# Supplementary material for: Predicting Treatment Response to Neoadjuvant Chemoradiotherapy in Rectal Mucinous Adenocarcinoma Using an MRI-Based Radiomics Nomogram
Source: Front Oncol. 2021 May 24;11:671636. doi: 10.3389/fonc.2021.671636 (PMC8181148; doi:10.3389/fonc.2021.671636)
Supplement: Supplementary file 1 [file Table_1.docx]

**Supplemental Table 1. Oblique axial high-resolution T2WI sequence parameters**

| Cohort | Scanner | Field Strength | Sequence name | Echo train length | TR/TE (m/s) | Matrix | FOV  (mm) | Section thickness (mm) | FA (°) | TA |
| --- | --- | --- | --- | --- | --- | --- | --- | --- | --- | --- |
| CH | SIEMENS | 3.0 T | TSE | 16 | 4000/108 | 320×320 | 180×180 | 3 | 160 | 4 min 10 sec |
|  | GE | 3.0 T | FSE | 32 | 7845/109 | 352×352 | 200×200 | 4 | 110 | 2 min 11sec |
| RJ | SIEMENS | 1.5 T | FSE | 15 | 4000/90 | 320×320 | 180×180 | 3 | 150 | 4 min 08 sec |

FA: Flip angle; FOV: Field of view; TA: Acquisition time; TR/TE: Repetition time/echo time; TSE: Turbo spin echo; FSE: Fast spin echo.

CH: Changhai Hospital.

RJ: RuiJin Hospital LuWan Branch.
